# One-stage posterior excision of lumbosacral hemivertebrae: Retrospective study of case series and literature review

**DOI:** 10.1097/MD.0000000000008393

**Published:** 2017-10-27

**Authors:** Yang Li, Guodong Wang, Zhensong Jiang, Xingang Cui, Tao Li, Xiaoyang Liu, Wen Zhang, Jianmin Sun

**Affiliations:** a Anatomical Institute of Minimally Invasive Surgery, Southern Medical University, Guangzhou; b Department of Spine, Shandong Provincial Hospital Affiliated to Shandong University, Jinan, Shandong Province, China.

**Keywords:** congenital scoliosis, hemivertebrae excision, lumbosacral hemivertebrae, one-stage posterior approach

## Abstract

Lumbosacral hemivertebrae causes unique problems as early trunk decompensation and long compensatory curve above. There are only a few reports on it. This case series is a fair supplement in the literatures.

To evaluate the clinical and radiological outcomes of lumbosacral hemivertebrae resection through 1-stage posterior approach.

Between 2005 and 2014, a consecutive series of congenital scoliosis due to lumbosacral hemivertebrae underwent hemivertebrae excision through 1-stage posterior only approach. Demographic, operative, radiological, and quality of life data were reviewed.

The mean lumbosacral curve was 29 ± 7° preoperatively, 10 ± 3° postoperatively, and 13 ± 5° at the final follow up. The final correction rate was 55 ± 9%. The gravity trunk shift was 11 ± 3 mm preoperatively, 37 ± 12 mm (range, 6–49 mm) postoperatively, 14 ± 9 mm at final follow up. The rib cage shift was 36 ± 12 mm preoperatively, 19 ± 5 mm postoperatively, and 15 ± 4 mm at the final follow up. The mean blood loss was 527 ± 125 mL and the mean surgery time was 336 ± 98 minutes. The mean follow up period was 41 ± 6 months. Two patients underwent transient neurological complications, 2 had wound bad healing, and 1 got wound infection. No pseudoarthrosis and instrumentation failure was observed.

One-stage posterior hemivertebrae excision could gain reasonable outcome. It is crucial to completely resect the hemivertebrae and the Y-shaped disc. Bending the rod to appropriate lordosis is helpful to close the convex side. Early surgical intervene is a preferred choice to restore the trunk balance and avoid extensive fusion. The neurological complication rate is high. Convex radiculopathy is often caused by retraction, it could recover at follow up.

## 1 Introduction

The lumbosacral hemivertebrae presents an unique problem,^[1]^ the absence of a mobile spine below the hemivertebrae leads to early truncal decompensation and a long compensatory curve above.^[2]^ Different from the lumbar hemivertebrae, the lumbosacral hemivertebrae usually does not present risk of kyphosis deformity.^[3]^ Single, fully segmented lumbosacral hemivertebrae leads to progressive scoliosis at a rate of 1.5° per year.^[4]^ Conventional methods as bracing or in site fusion are useless.^[2,5]^ Thus, excision of the lumbosacral hemivertebrae is usually recommended.^[6]^

The lumbosacral hemivertebrae resection is usually operated through combined anterior and posterior approach.^[2,7]^ It is reported to be effective and with fair outcomes at long-term follow up.^[2,3,6]^ Zhang et al reported at 2015 a case series of lumbosacral hemivertebrae resection through posterior only approach.^[8,9]^ The procedure was successfully performed on 14 cases and the midterm follow up outcome was fairly well.^[8,9]^ However, there's no other large case series report on it.

The aim of our retrospective study is to review 12 consecutive cases enrolled in our department, who underwent lumbosacral hemivertebrae resection through 1 stage^[10]^ posterior approach, in order to evaluate the clinical and radiological outcomes and provide a supplement in the literature.

## 2 Materials and methods

Permission to conduct this retrospective study was obtained from the hospital ethics committee.

Since 2005, the senior author has used the posterior only approach to perform lumbosacral hemivertebrae resection. Between January 2005 and December 2014, 12 consecutive congenital scoliosis patients due to lumbosacral hemivertebrae were included in this study. They all underwent hemivertebrae excision and fusion through 1-stage posterior only approach. All the surgeries were performed by the same surgeon in a university affiliated hospital.

Inclusion criteria: congenital scoliosis due to lumbosacral hemivertebrae, hemivertebrae excision performed through posterior approach, with follow up more than 2 years.

Exclusion criteria: non-congenital scoliosis, without lumbosacral hemivertebrae, hemivertebrae excision performed through combined approach or anterior approach, follow up period <2 years.

### 2.1 Preoperational evaluation

Detailed physical and neurological examinations were performed. Preoperational radiological imaging included full spine anteroposterior (AP) and lateral radiographs, dynamic radiographs including lateral bending, extreme lumbar flexion and extension, three dimensional computer tomography scan (CT), and magnetic resonance imaging (MRI). All children underwent echocardiography and ultrasound of the urinary tract before surgery to assess associated congenital abnormalities. Scoliosis Research Society-22 (SRS-22) questionnaire was used to evaluate the health-related quality of life as the baseline before surgery.^[10]^ Demographic data were recorded including sex, height, body weight, age, and et al.

### 2.2 Operation data

All the cases underwent 1-stage hemivertebrae excision and fusion through posterior only approach. During the surgery, spinal cord monitoring and wake up test were used to prevent neurologic complications. Spinal cord monitoring included somatosensory evoked potential (SEP), motor evoked potential (MEP), and motor spontaneous potential. Detailed surgery information was recorded. It included operation time, blood loss, blood transfusion, level of fusion, information of spinal cord monitoring, and et al.

### 2.3 Postoperation and follow up

All the patients were followed up every half year. Full spine anteroposterior and lateral radiographs were taken after the surgery and at every follow up. Detailed physical and neurological examinations were also performed. Scoliosis Research Society-22 (SRS-22) questionnaire was used to evaluate the health-related quality of life at every follow up.

### 2.4 Radiological parameters

Full spine radiographs were reviewed to record the location of the hemivertebrae, the segmental curve, the main curve, the compensatory cranial curve, the trunk shift, the segmental kyphosis, and the overall sagittal alignment. The segmental curve was measured between the superior plate of the 2 vertebrae adjacent to the hemivertebrae. The main curve was the maximal curve angle of the lumbosacral scoliosis. Trunk shift was evaluated by 2 different methods: the gravity trunk shift and the rib cage shift. The gravity trunk shift was the distance between a plumb line drawn from the middle of C7 body and the middle of sacrum. The rib cage shift was calculated by Luk method.^[2]^ A perpendicular line was drawn at the most convex aspect of the outer pelvic border to a line joining the top of the iliac crests, then a line drawn parallel to this perpendicular line at the most laterally displaced aspect of the rib cage was used to measure the maximum rib cage translational shift. On the lateral view, segmental kyphosis was measured as the angle between the superior plate of the 2 vertebrae adjacent to the hemivertebrae. The global lumbar lordosis was between superior plate of L1 and superior plate of S1. The thoracic kyphosis was between superior plate of T5 and superior plate of T12. A kyphosis curve was expressed as positive angle values, whereas negative values correspond to lordotic curves.

### 2.5 Clinical outcomes

The quality of life data was evaluated using SRS-22 questionnaire. The questionnaires were completed by the children themselves if they were older than 12 years, otherwise were completed by their parents. Total scores as well as individual domain scores for pain, self-image, function, mental health, and satisfaction parameters were calculated and analyzed for each patient.

One author as the independent observer finished the evaluation procedure in a blinded manner. Statistical data were analyzed using Statistical Package for Social Sciences (SPSS, version 20, IBM) software. Comparison between preoperation, postoperation, and the final follow up was explored with ANOVA or Pearson Chi-square test for the demographic data, the SRS-22 scores, and the radiological parameters. *P* values <.05 was regarded as statistically significant and two-sided tests were used during all the analyses.

## 3 Results

Among all the 12 lumbosacral hemivertebrae cases, there were 7 girls and 5 boys. The average age was 7.2 ± 1.9 years old (range, 5–12 years old). Among the 12 lumbosacral hemivertebrae, there were 7 full segmented and 5 semi-segmented. One case associated with lumbar hemivertebrae, 2 cases with thoracic vertebral anomalies, 3 cases with syringomyelia, 1 case with split cord malformation. No neural deficit was observed before the surgery. The radiographic characters of every lumbosacral hemivertebre could be seen in Table 1.

**Table 1:**
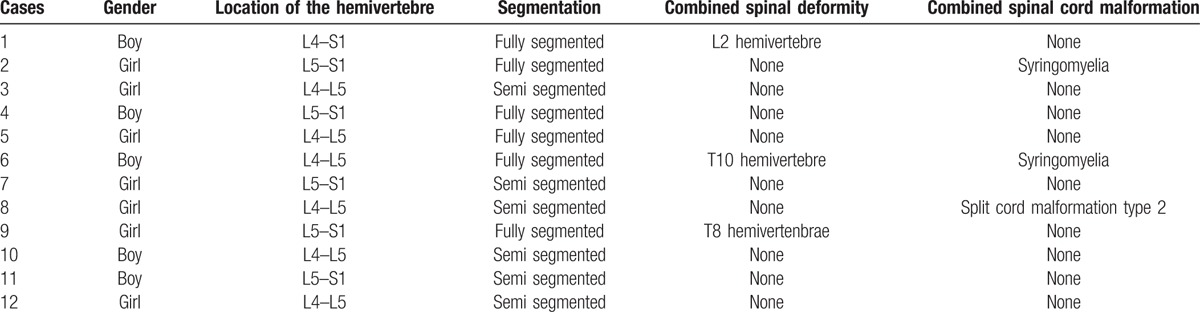
The radiographic characters of the lumbosacral hemivertebres.

The lumbosacral hemivertebrae was excised and fused through 1-stage posterior-only approach by 1 senior author. The mean fusion level was 2.7 ± 1.0 segments (range, 2–5 segments). The mean operation time was 336 ± 98 minutes (range, 212–437 min), the mean blood loss was 527 ± 125 mL (range, 210–1400 mL). The mean follow up time was 41 ± 6 months (range, 29–63 months).

**Figure 1:**
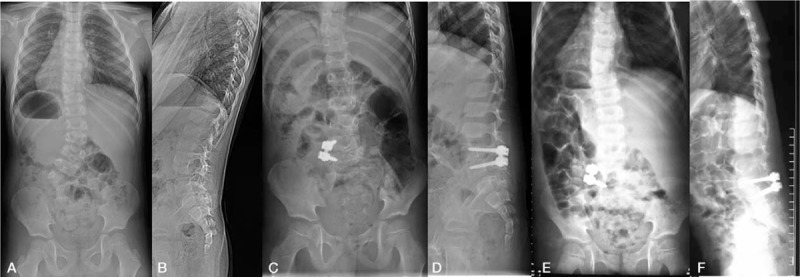
The patient was a 5-year-old boy with L5 hemivertebrae. One-stage posterior only approached hemivertebrae excision was performed. Reasonable radiological outcome was observed at follow up. A and B: Preoperative full spine radiographs showed the segmental curve was 43°, the compensatory lumbar curve is 22°. The coronal trunk balance was good. C and D: postoperative full spine radiographs showed the segmental curve was 8°, the compensatory lumbar curve is 15°. E and F: the full spine radiographs at final follow up showed the segmental curve was 9°, the compensatory lumbar curve is 13°.

The segmental curve was 29 ± 7° (range, 21–45°) before the surgery, 10 ± 3° (range, 8–22°) after the surgery, and 13 ± 5° (range, 9–26°) at the last follow up. An example could be seen in Fig. 1. The final correction rate was 55 ± 9% (range, 21–85%). The compensatory lumbar curve was 34 ± 10° (range, 22–52°) preoperatively, 14° (range, 7–24°) postoperatively, and 12° (range, 8–25°) at the last follow up. The segmental lordosis was 12° (range, 10–18°) preoperatively, 21° (range, 17–29°) postoperatively, and 17° (range, 13–28°) at final follow up. The gravity trunk shift was 11 ± 3 mm (range, 4–18 mm) before the surgery, 37 ± 12 mm (range, 6–49 mm) after the surgery, 14 ± 9 mm (range, 4–22 mm) at the final follow up. The rib cage shift was 36 ± 12 (range, 21–50 mm) preoperatively, 19 ± 5 mm (range, 10–28 mm) postoperatively, and 15 ± 4 (4–21 mm) at the final follow up. sagittal vertebral axis was 9 ± 8 mm (range, −10 to 25 mm) preoperatively, 19 ± 8 mm (range, 10–40 mm) postoperatively, and 10 ± 5 mm (range, −5 to 20 mm) at the final follow up.

All the patients completed the SRS-22 questionnaires. The total SRS-22 score was 3.6 ± 0.3 before the surgery, 4.1 ± 0.4 at the last follow up. It demonstrated statistically significance with the *P* value as .01.

### 3.1 Complications

There were 3 patients with neurological complications. Two patients underwent dorsal extensor paralysis and L5 nerve radiculopathy at the convex side. The other one underwent hyperalgesia at the distribution of convex S1 nerve. The neurological complications occurred at 3 days after the operation and recovered at the 7 to 9 days after the operation. Short acting glucocorticoid, such as methylprednisolone, was administrated. No surgical intervene was performed.

There were 2 patients with incision site bad healing. Insidious subcutaneous tissue lysis and resewing were performed. One patient was diagnosed as early infection. Vancomycin was administrated for 2 weeks until the laboratory test became normal. No pseudoarthrosis was observed. No instrumentation failure was observed.

## 4 Discussion

The lumbosacral hemivertebrae is a challenging problem. The primary goal of the treatment is to prevent the development of severe segmental deformity, early trunk shift, and long compensatory curve above,^[8,11,12]^ due to absence of a mobile spine below the lumbosacral hemivertebrae.^[1,13]^ The lumbosacral hemivertebrae resection through combined anterior and posterior approach is usually recommended with fair outcomes at long-term follow up.^[2,5–7]^ The lumbosacral hemivertebrae excision through posterior only approach is a newly proposed procedure.^[8,9]^

According to previous reports, when compared with lumbar and thoracolumbar hemivertebrae, the lumbosacral hemivertebrae spends more operation time, causes higher neurological complication rate, and turns out with poorer correction.^[11]^ There are several aspects affecting the radiological and clinical outcomes, including segmental deformity correction, trunk shift, and overall alignment and neurological complications.

### 4.1 Segmental deformity correction

If the lumbosacral hemivertebrae was left untreated, the compensated lumbar curve would have progressed 2 times quicker than the lumbosacral curve.^[1]^ Bracing cannot prevent the lumbosacral curve deteriorating.^[5]^ Thus, surgical intervene is recommended.

Despite the Cobb angle of lumbosacral curve is relatively small, the correct rate is not quite good. The segmental curve is 30 to 35° according to previous report, the correction rate is 20% when the lumbosacral hemivertebrae is not removed and 56% to 85% with the hemivertebrae resected.^[1–3,8,12,14,15]^ In our study, the segmental curve is 28.7° and the correct rate is 55 ± 9% (range, 21–85%).

The segmental curve correction depends on the resection of the lumbosacral hemivertebrae (HV).^[2]^ It can be resected through both antero-posterior combined approach and posterior-only approach.^[7,9]^ All the lumbosacral HVs are removed through 1-stage posterior approach in the present study. The radiological outcome shows the segmental curve correction rate is comparable to the previous reports.^[8]^

There are 3 critical points to gain great correction rate.^[2]^ The first one is to remove the whole HV as well as the Y-shaped intervertebral disc. The residual tissue and disc at the concave side would hinder the convex closure. The second one is that the rod should be pre-bended to an appropriate angle. Because of the large lumbosacral lordosis, a relatively straight rod would do harm to the correction. The last one is to avoid using cage to fulfill the convex cavity which is supposed to close.

### 4.2 Trunk shift and overall balance

Due to the location of lumbosacral HV, it affects the pelvis base more than lumbar and thoracic HV.^[15]^ Thus, it caused early truncal decompensation and long compensatory curve above. When the lumbar compensative curve becomes structural, the trunk shift would be hard to handle.^[16]^

The overall balance can be achieved through lumbosacral HV resection in early age. The trunk shift correction is 55% according to Luk report.^[2]^ However, there are over one-fourth patient with unlevel lower limbs, which should be taken in charge when evaluating the trunk balance. If the lumbar compensative curve is >40° or associated with lumbar HV, the 2 curves both need to be fused.^[8]^

Among all the 12 patients in the present study, 10 patients underwent short fusion. There is 1 patient with lumbar HV and another one with the lumbar curse as 52°. The 2 patients underwent long fusion from lumbar spine to sacrum. Due to no residual mobile segment below, the balance should be restored during the surgery. In-operation radiograph is helpful to assess the overall balance. Fluoroscopy through C-arm machine cannot cover the pelvis and lumbar spine.

### 4.3 Neurological complications

The neurological complications vary widely according to previous reports. In Leong report, the rate was 1 out of 6.^[2]^ They pointed out that the neurological complication is not inevitable and can relieve well without intervene.^[2]^ There was only 1 patient with transient neurological complication in Zhang study.^[8]^ Bollini et al^[3]^ reported the nerve injury rate was 5.6%. There was no permanent neurological complication.^[1]^ It was mainly caused by retraction at the convex side.^[2]^ In the present study, there are 3 patients with convex nerve radiculopathy. No revision surgery is needed. Short acting glucocorticoid, such as methylprednisolone, could improve the radiculopathy. Dexamethasone as long acting glucocorticoid should be avoided since it inhibits the growth and chondrotropic hormone. The complications recover eventually at follow up.

Spinal cord monitoring is helpful to prevent neurological complications. Dermatomal somatosensory evoked potential (DSEP) and motor evoked potential (MEP) could locate the specific nerve root and monitor it during the surgery. However, most of the neurological complications are delayed nerve injury, often occur at 3 days after surgery. Spinal cord monitoring usually shows no positive alert.

Besides the neurological complication, the incision site complications should be paid attention on. It includes wound bad healing and surgical site infection. The wound bad healing is usually caused by excessive tension on the incision site skin. Children often have thin back muscle, thus low profile screws are preferred than the high profile ones. Insidious subcutaneous tissue lysis is very helpful to release the tension of incision site. Surgical site infection is another cause bad healing. Early diagnosis and intensive antibiotics ministration can cure the infection before needing debridement.
